# *Lasius fuliginosus* Nest Carton as a Source of New Promising Bioactive Extracts with Chemopreventive Potential

**DOI:** 10.3390/ijms22094392

**Published:** 2021-04-22

**Authors:** Grzegorz Karol Wagner, Magdalena Jaszek, Bernard Staniec, Monika Prendecka, Dominika Pigoń, Anna Belcarz, Dawid Stefaniuk, Anna Matuszewska, Ewa Pietrykowska-Tudruj, Mirosław Zagaja

**Affiliations:** 1 Department of Zoology and Nature Protection, Institute of Biological Sciences, Maria Curie-Skłodowska University, Akademicka 19, 20-033 Lublin, Poland; bernard.staniec@poczta.umcs.lublin.pl (B.S.); ewa.pietrykowska@umcs.pl (E.P.-T.); 2 Department of Biochemistry and Biotechnology, Institute of Biological Sciences, Maria Curie-Skłodowska University, Akademicka 19, 20-033 Lublin, Poland; dawid.stefaniuk@umcs.pl (D.S.); anna.matuszewska@poczta.umcs.lublin.pl (A.M.); 3 Chair and Department of Human Physiology, Medical University of Lublin, Radziwiłłowska 11, 20-080 Lublin, Poland; monika.prendecka@umlub.pl (M.P.); dominika.pigon@umlub.pl (D.P.); 4 Chair and Department of Biochemistry and Biotechnology, Medical University of Lublin, Chodźki 1, 20-093 Lublin, Poland; anna.belcarz@umlub.pl; 5 Isobolographic Analysis Laboratory, Institute of Rural Health, Jaczewskiego 2, 20-090 Lublin, Poland; zagaja.miroslaw@imw.lublin.pl

**Keywords:** jet black ants, carton derived extracts, biochemical analysis, FTIR spectra, antioxidative capacity, chemopreventive effect, anticancer effect, melanoma cells

## Abstract

Six new water extracts (E1–E6) were obtained from nest carton produced by jet black ants *Lasius fuliginosus* and tested for their biochemical and bioactive properties, including antioxidative and anticancer effects. The present study demonstrated significant qualitative and quantitative differences in the content of individual biochemical constituents, as well as bioactive properties between the investigated samples. All tested extracts demonstrated antioxidant properties (determined using 2,2-diphenyl-1-picrylhydrazyl (DPPH) and 2,2’-azino-bis(3-ethylbenzothiazoline-6-sulfonic acid (ABTS) methods), and the highest antioxidative potential was recorded in extracts E1 and E2 (188.96 and 313.67 μg/mL of ascorbic acid equivalent for ABTS and 176.42 and 202.66 μg/mL for DPPH reagent). Furthermore the six extracts exhibited strong inhibitory activity towards human melanoma cells of the A-375 CRL-1619 line in a dose-dependent manner. The most interesting chemopreventive activity was exhibited by extract E2, which inhibited the proliferation of A-375 cells to the greatest extent, while having a minimal effect on Vero cells. The effect on cancer cells has been confirmed using the Electric Cell-substrate Impedance Sensing (ECIS) technique. Significant impedance changes have been detected in A-375 and Vero cells following the administration of extract E2. The obtained results are really promising and constitute the basis for further research on the nest carton of jet black ant.

## 1. Introduction

For centuries, nature has been the source of many bioactive products used by humans; at first, these were made from plants or fungi. Neolithic peoples were familiar with methods of producing medicines, oils, pigments, etc., from different species of plants, fungi, or their parts [1,2]. Nowadays, the requirements of contemporary medicine, including the highly dynamic increase in the incidence of civilization diseases, continue to pose new challenges to science. More and more natural substances with beneficial medicinal properties have come into use. In the late 19th century, bioactive products were extracted from the white willow *Salix alba*, on the basis of which acetylsalicylic acid was synthesized soon after, bringing fame to the Bayer pharmaceutical company. In subsequent years, the number of discoveries increased, with substances from organisms other than plants also being looked at. A discovery that revolutionized medicine was the isolation of penicillin from the *Penicillium* fungus in 1938, for which Fleming, Florey, and Chain were awarded the Nobel Prize some years later [3]. The demand for this kind of knowledge created a favorable climate for the development of such sciences as chemistry, which aimed to discover the compositions of substances not only of plant origin, but also of animal and mineral provenance [4]. Nowadays, the search for bioactive substances of natural origin is proceeding apace. Apart from studies of substances obtained from plants, the literature boasts numerous papers describing research into bioactive substances produced by fungi and insects. The results of studies of substances produced by fungi associated with insects seems to be interesting with respect to their biological activity, including anticancer properties. A number of such analyses relate to entomopathogenic fungi. Worth mentioning in this context is the work by Xiao et al. [5], concerning secondary metabolites from the genus *Cordyceps* and their antitumor activity with very wide, multi-target, multi-level, and multi-pathway characteristics, and also the work by Patocka [6] on the entomopathogenic fungus *Beauveria bassiana*, as a result of which an alkaloid with a high neuroprotective potential was extracted, which could find application in combating and treating many neurodegenerative diseases. Interesting results have been obtained from research into substances derived from insects, especially ants. For instance, Arbiser et al. [7] published a promising study in which they demonstrated that solenopsin A, the main alkaloid in the venom of fire ants *Solenopsis invicta*, exhibits anti-angiogenic activity. Another example comes from the work of Sen et al. [8], who isolated strains of the bacteria *Pseudonocardia* and *Amycolatopsis*, which display distinct anti-fungal activity, from the nests of harvester ants (Attini) and from the ants themselves. The results of work by Hasanuddin et al. [9] are likewise promising: from material taken from the ants nests in plant *Myrmecodia pendens* in Papua New Guinea, they extracted a bioactive terpenoid and demonstrated its antitumor activity. This constituent induced apoptosis of ovarian cancer cells of the SKOV-3 line by activating caspase-9.

Very interesting microbiological studies have also been carried out on jet black ants *Lasius fuliginosus*: from the heads of these ants Lan Ye et al. [10] extracted a hitherto unknown strain of the actinobacteria *Streptomyces lasiicapitis*, which produces canchanomycin, a substance with antifungal activity. Krishanti et al. [11] carried out a similar study on termite nests. These researchers discovered a series of actinobacteria strains that produce antimicrobial substances, thereby demonstrating that termite nests are a new source of bactericidal substances with a high therapeutic potential. The results of a two-year study by Brinker et al. [12] of fungi and bacteria from the carton of *L. fuliginosus* nests and the surrounding soil are interesting. These authors demonstrated the high stability of the nest’s mycoflora and its similarity to the fungal assemblages in the surroundings, as well as the fluctuations in the structures of bacterial assemblages and their differentiation between the nest and its surroundings. Moreover, they demonstrated the inhibitory influence of preparations made from ants towards the pathogenic fungus *Metarhizium* sp. Interestingly, when an extract of ants along with fungi from the nest was incubated, not only was there no inhibition—the mycelium actually grew faster than in the control sample. This testifies to the long-term co-evolution of an active mechanism enabling jet black ants to manage microbe assemblages, probably governed by the host’s secretions.

To date, however, no studies have been carried out on the synergistic biological activity of an extract obtained from the nest carton (nest building material) of jet black ants. Hence, our main aim was to produce extracts of this carton obtained from different natural habitats and then to assess its biochemical properties, composition, and bioactivity, in particular its antioxidative potential and effects on human malignant melanoma cells. The most commonly used measurement methods based on spectrophotometric detection were used to determine antioxidant properties: the method using the radical ABTS+, i.e., 2.2-azinobis-(3-ethylbenzothiazoline-6-sulfonate), and the radical DPPH^•^ (2.2-diphenyl- 1-picrylhydrazyl). These methods, thanks to their simplicity and efficiency, are very convenient tools for assessing the antioxidant power of many bioactive preparations [13]. 

## 2. Results

### 2.1. Characterization of the Ant Nest Structure 

**Figure 1:**
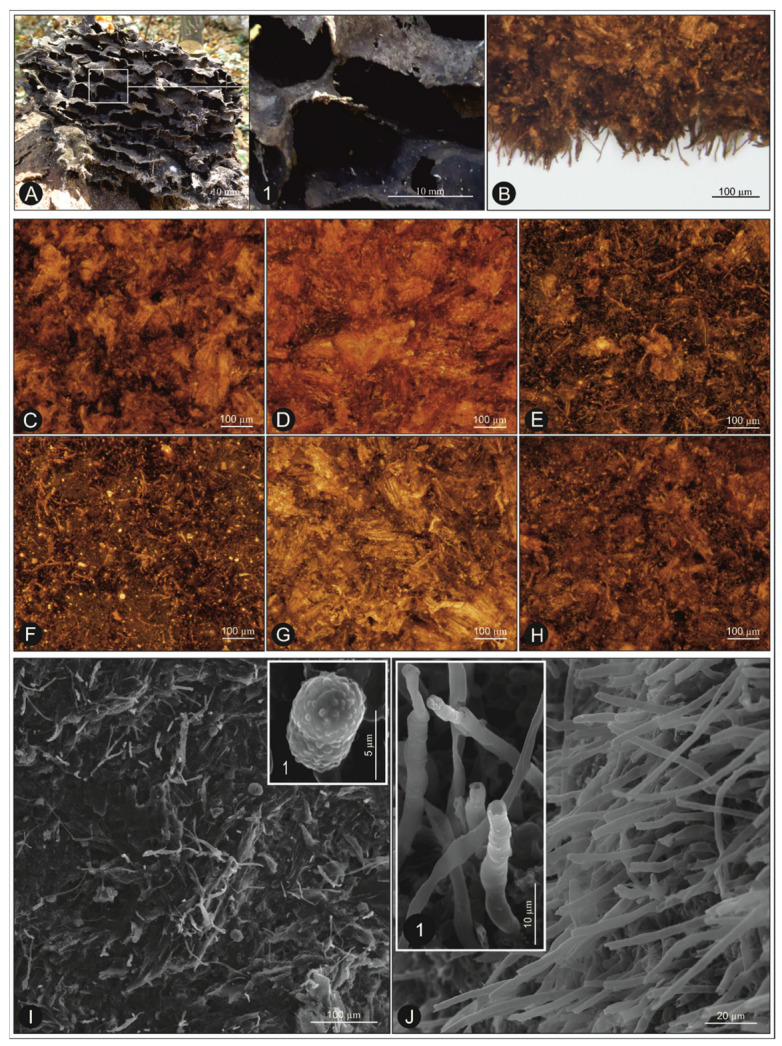
The carton nest taken out of the hollow in the field (forest near Długie Lake, Polesie National Park, Poland) (**A**); macrophotography of the chambers in the nest (**A1**); the edge of carton with visible fungal structures (**B**); structure of carton from different nests which were the basis of extracts E1–6 (**C**–**H**, respectively); microstructure of carton (**I**,**J**) with spores; (**I1**) and fungal structures (**J1**) (phot. (**A**–**H**): G. K. Wagner; phot. (**I**,**J**): B. Staniec).

The carton from each of the six ant nests was different in color, granulation of wood material, and the proportion of individual fractions included in it (wood, fungal structures, binder-ground wood material mixed with ant secretions and honeydew) (Figure 1C–H). In addition, there were differences in the structure of the carton within the same nest, possibly due to different levels of humidity and various proportions of mycelium. However, the overall construction plan was always the same. The medium prepared by ants on the basis of wood particles was more or less covered with mycelium hyphae (Figure 1I–J), between which spores were visible. The microsculpture of the spores is shown in Figure 1I1. 

### 2.2. General Properties of the Ant Nest Extracts 

#### 2.2.1. Total Carbohydrates (TC), Proteins, Phenolic Compounds (PC), Relative Level of Superoxide Anion Radicals (SOR), and Proteolytic Activity Analysis 

**Figure 2:**
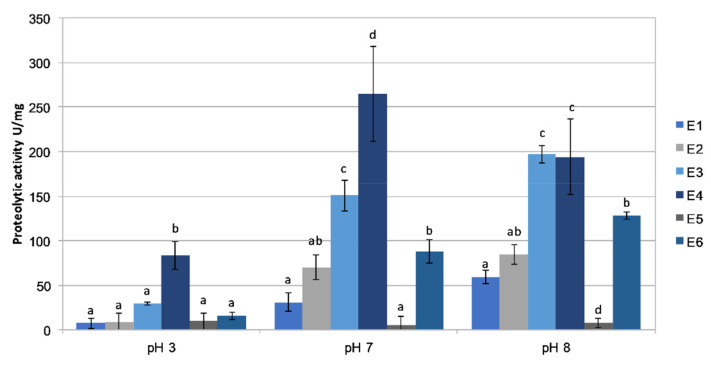
The specific protease activities of the ant nest-carton preparations E1–6 tested in three pH ranges: 3.0, 7.0, and 8.0. The means marked with different letters are significantly different (*p* ≤ 0.05).

Biochemical analysis of extracts E1–6 revealed significant qualitative and quantitative differences in the content of individual constituents between the samples tested. The Bradford method showed the highest concentration of protein to be in samples E1 (97 μg/mL) and E2 (92.2 μg/mL), and the lowest in E4 (27.12 μg/mL). The protein levels in the other samples were at a similar level, from 78.9 to 83.3 μg/mL (Table 1). The concentration of phenolic substances (PC) (DASA test) was the highest in sample E2 (0.19 mM) and the lowest in E4 (0.05 mM); the latter extract was of material obtained from a subterranean ant nest situated under a silver birch (*Betula pendula*). The PC levels in samples E1, E3, and E5 were at a similar level: 0.13, 0.14, and 0.14 mM, respectively (Table 1). The highest total carbohydrate (TC) level was detected in sample E2 (0.241 mg/mL), whereas the other five extracts (E1, E3–E6) contained from 0.145 to 0.17 mg of TC per mL (Table 1). Analysis of the SOR content in the samples showed that the highest relative level was present in E1 and E5, and the lowest level in E4 (Table 1). Enzymatic analysis of the extracts showed that proteolytic enzymes were active in all the extracts. Protease activity was the highest in samples E3 and E4 in all the pH ranges (pH 3.0, 7.0 and 8.0) (Figure 2). In all cases, the proteolytic activity of the extracts was much higher at pH 7.0 and pH 8.0 than at pH 3.0. Protease activity was the highest in samples E3 and E4 (151.1 and 264.5 U/mg of protein at pH 7.0, and 197.3 and 194.1 U/mg at pH 8.0, respectively). A relatively high level of substrate digestion was also observed in the case of extract E6 (87.7 U/mg of protein at pH 7.0 and 128.1 U/mg at pH 8.0 (Figure 2)).

#### 2.2.2. Fourier-Transform Infrared Spectroscopy (FTIR) Analysis of Samples

**Figure 3:**
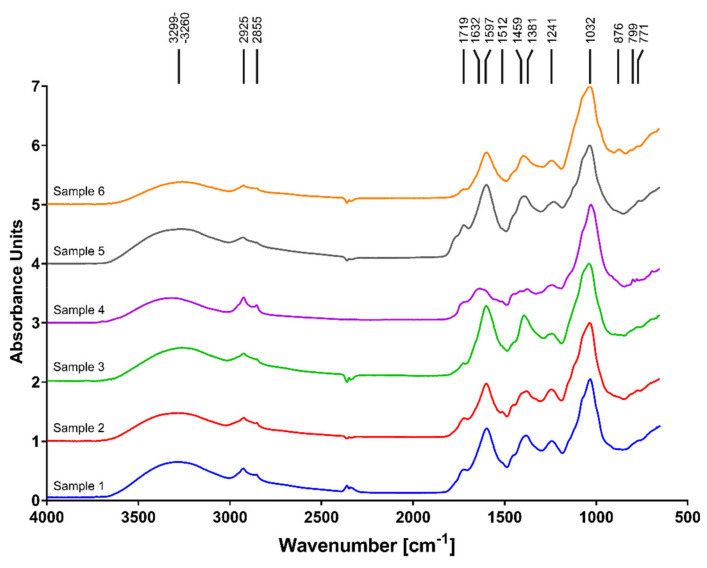
FTIR-ATR spectra of the aqueous ant nest extracts E1–E6 (samples 1–6, respectively).

The FTIR spectra of the six aqueous extracts (E1–6) showed broad peaks (Figure 3); this is quite typical of complex organic extracts, in contrast to pure substances, which have rather narrow, sharp peaks. Peaks characteristic of polysaccharides were observed in all the samples: the broad peak at 3100–3500 cm^−1^ may be due to -OH stretching vibrations; the peaks at 2920–2935 cm^−1^ and 2855 cm^−1^ to antisymmetric and symmetric stretching vibrations of = CH_2_ and -CH_3_ groups, respectively; those at 1241 cm^−1^ and 1032 cm^−1^ to C-O-C stretching vibrations; and the peak at 771 cm^−1^ to C-C stretching vibrations [14]. In addition, the spectrum of extract E6 shows an absorption band at 876 cm^−1^ characteristic of a pyranoside ring (C-H ring vibration) [15]. The bands at 3100–3500 cm^−1^, 2920–2935 cm^−1^, and 2855 cm^−1^ are also characteristic of lignin-derived phenolic compounds like vanillyl, syringyl, and cinnamyl units (acids and aldehydes), as are the –CH_3_ symmetric stretching vibrations at 1459 cm^−1^ [16]. These bands are all present in extracts E1–6. The bands at 1719 cm^−1^ and 1597 cm^−1^ are due to the presence of C = O stretching vibrations in carboxylic groups. The 1719 cm^−1^ band may suggest the presence of uronic acids in sugars or phenolic acids resulting from lignin decay. Additionally, the spectra of all the extracts E1–6 showed peaks at 1719 cm^−1^ and 1241 cm^−1^, which suggest the presence of o-acetyl esters in sugars [15]. The band at 1632 cm^−1^, observed in sample E4, may be attributed to bound water [17], to stretching vibrations of C = C bond in phenolic compounds like vanillyl or syringyl units [18] or to an amide I band. This last possibility is enhanced by the presence of a very weak 1538 cm^−1^ band (amide II) in E4; this extract may also contain sulphate esters of hexose, as shown by the presence of C-O-S vibrations at 799 cm^−1^ [19]. The spectrum of extract E2 exhibited the presence of a band at 1512 cm^−1^, which may be attributed to –C = C- stretching vibrations in the aromatic ring in metal-phenolic acid complexes, as demonstrated for Fe (III)-syringic acid complexes [18].

### 2.3. Biological Properties of the Water Ant Nest Extracts

#### Total Microbial Activity of the Samples (TMA)

Determination of the total microbial activity (TMA) of the extracts showed the highest level of released fluorescein (mg/kg/h) in extract E6 (0.61). The values recorded for E3–5 were at a similar level—0.56, 0.55, and 0.46, respectively (Table 1). Extract E2 took the lowest value of TMA (0.37) (Table 1). 

### 2.4. Antioxidant Capacity

Although all the extracts (E1–6) demonstrated antioxidant properties (determined by DPPH and ABTS), there were significant differences in antioxidant level between them. The highest antioxidative potential of ABTS was recorded in extracts E1 and E2 (188.96 and 313.67 μg/mL of ascorbic acid equivalent, respectively). The same correlations were obtained using DPPH (176.42 and 202.66 μg/mL of ascorbic acid equivalent, respectively). The scavenging effect was the least for extract E4 (96.12 with ABTS and 98.75 μg/mL with DPPH) (Table 1).

### 2.5. Cytotoxic and Antiproliferative Effects of the Extracts

**Figure 4:**
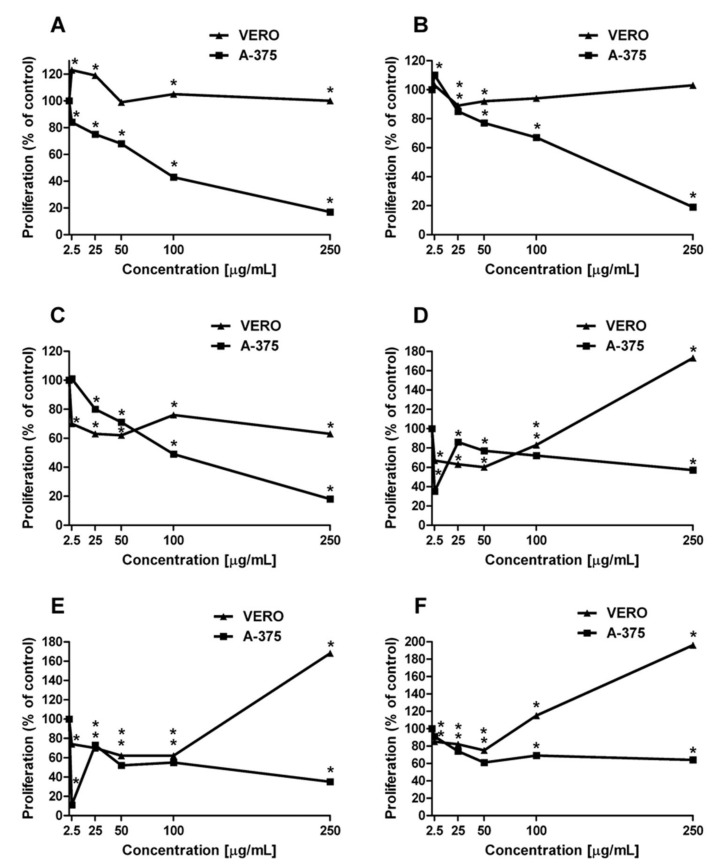
Inhibition of proliferation of A-375 and Vero cells by extracts E1 (**A**), E2 (**B**), E3 (**C**), E4 (**D**), E5 (**E**), and E6 (**F**), measured by MTT. Values are expressed as a percentage of the control regarded as 100%; * *p* < 0.01, ** *p* < 0.005, one-way Anova, Dunnett’s test.

All the extracts inhibited cell proliferation in a dose-dependent manner. Nevertheless, all of them elicited the most pronounced effect after 72 h of incubation. As the statistically significant effect did not manifest itself until after this period, the effect is antiproliferative rather than cytotoxic. The activity was stronger towards cancer cells as the proliferation of A-375 cells was reduced to 17, 19, 18, 57, 35, and 64% of the control by extracts E1, E2, E3, E4, E5, and E6 at 250 μg/mL, respectively (Figure 4). 

Extracts E1, E2, E3, and E6 at 100 μg/mL concentration still had a significant impact on melanoma cell viability—43, 67, 49, and 69%, respectively, in comparison to the Vero cells. Lower concentrations (2.5, 25, 50 μg/mL) of all the extracts had no significant effect on A-375 cancer cells. The tested extracts had no inhibitory effect on healthy Vero cells; on the contrary, they improved viability compared to control cultures (Figure 4). The most desirable activity was exhibited by extracts E2, E4, and E5, i.e., the proliferation of A-375 cells was inhibited to the greatest extent, while having the mildest effect on the Vero cells (Figure 4). The IC_50_ values of extracts E1-E6 was also determined in the present study (Table 2). 

#### Effect of Extract E2 on A-375 and Vero Cells as Recorded by Electrical Parameter Measurements

The effect of 250 µg/mL of E2 on A-375, the Vero monolayer and the corresponding changes in electrical parameters was monitored continuously for 90–120 h. Using the ECIS system, we recorded important changes in impedance in A-375 and Vero cells following the administration of extract E2. The results are presented in the form of figures, where the error bars are plus and minus one standard deviation and are plotted at regular intervals along the graph. 

**Figure 5:**
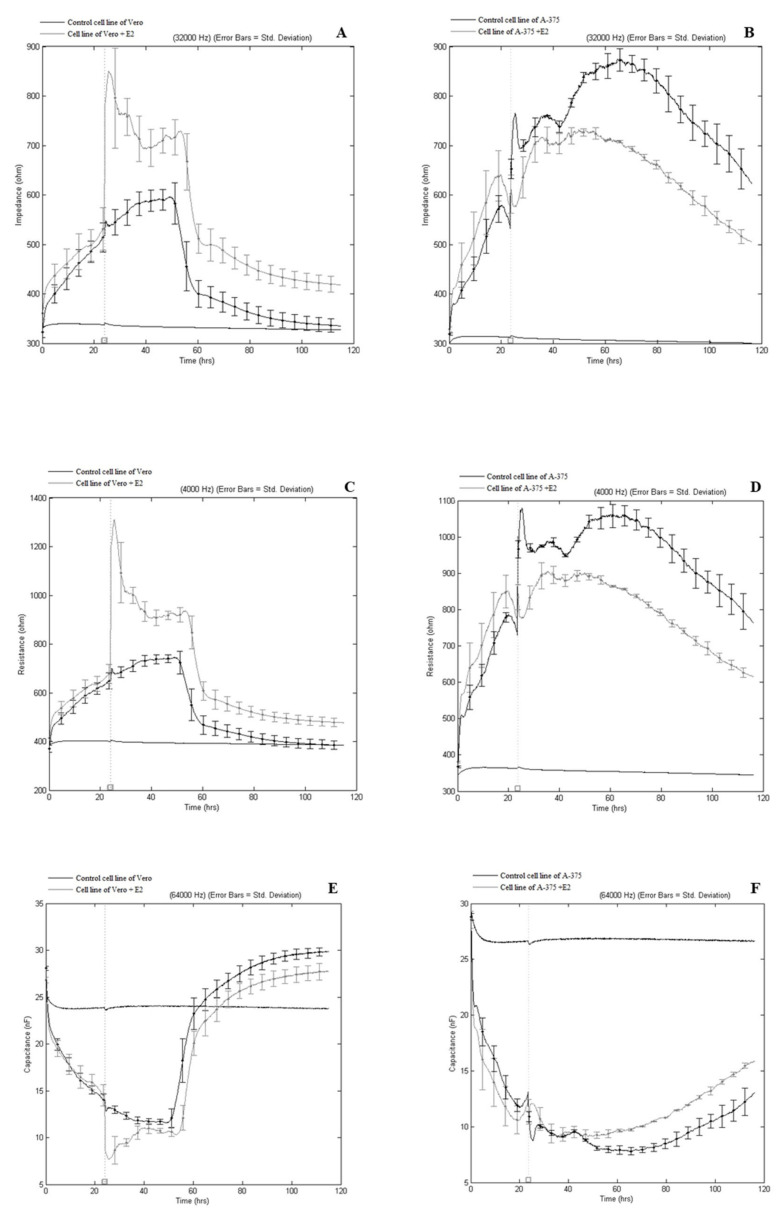
Changes in impedance (**A**,**B**), resistance (**C**,**D**), and capacitance (**E**,**F**) of Vero (**A**,**C**,**E**) and A-375 (**B**,**D**,**F**) cells treated with extract E2.

Based on the results, one can state that the test substance affected the electrical parameters during cultivation in different ways (Figure 5A–F). Every cell type has its characteristic adhesion and growth curve that can be manipulated by, for example, stimuli like chemical structure or the concentration of substances in the medium. The direct parameters derived from the impedance measurements are the resistance and capacitance of cells. The quality and function of the cell barrier is represented by the resistance, so the resistance towards para- and trans-cellular current flow should be considered. The capacitance provides an overall measure of electrode coverage. 

The different behavior of the cells after seeding, adherence, and proliferation, as well as their reaction to substances added to the medium, thus cause changes in impedance. Based on impedance, resistance, and capacitance measurements in the cultures of the cells exposed to E2, there are significant differences in the impedance values, indicating the different effects of E2 on these cells. A very interesting effect was recorded in the case of the Vero cells. Initially, even before the preparation was administered, the impedance slowly increased in all wells up to a value of about 500 ohms during ca 24 h, indicative of cell proliferation (Figure 5A). After this time, the preparation was administered. The impedance in the Vero cell cultures rose to 700 ohms between 25 and 50 h of the experiment, after which it gradually dropped. It is interesting that these values were significantly lower in cultures not treated with the extract (Figure 5A), which may even suggest a nutritional effect of the preparation on these cells. The increase in impedance is explained by the increase in cells and adhesion to the electrode of an increasing number of cells (this increases the resistance of the conductor, which is expressed as an increase in impedance). Its decrease indicates death of cells and their detachment from the electrode, thereby reducing electrode resistance. An increase in impedance indicating cell adhesion and proliferation accompanies a decrease in capacity, which was also demonstrated in this study.

An analogous experiment was carried out for human melanoma cells of the A-375 CRL-1619 line. Figure 5B,D,F show that the test extract had different effects on A-375 cells and Vero cells. In the case of the A-375 line, significantly lower impedances were recorded in the cultures treated with E2 compared to the control cultures, which indicates the potentially promising antitumor activity of the test extract. E2 had a particularly strong effect on A-375 cells (Figure 5B,D,F), which was determined on the basis of the impedance (Figure 5B) and resistance (Figure 5D) differences obtained in this culture. In the case of the control cultures, the highest impedance (nearly 900 ohms) was found between 50 and 80 h of the experiment (Figure 5B). Significantly lower impedances were recorded (nearly 700 ohms) in the same hour range in A-375 cells treated with E2, which indicates the potential antiproliferative effect of E2 on these cells.

Both the decrease in capacitance and the increase in impedance indicate cell proliferation, which is why the two values complement each other. The ECIS data (Figure 5E) showed that the capacitance of the Vero cultures decreased the most compared to the other cell lines, reaching the lowest value of about 10 nF between 30 and 60 h, after which it increased significantly to nearly 30 nF in both the control and Vero cells treated with E2 (Figure 5E). However, the still higher capacitance in the Vero control cells in comparison to the cells treated with E2 indicates that the preparation has a protective effect on the former. The opposite effect was observed in the case of capacitance measurements made in the control cultures and cultures of A-375 cells treated with E2 (Figure 5F). The higher capacitances in the cultures with the test preparation indicate its possible antiproliferative potential towards A-375 cells.

## 3. Discussion

The problems of modern medicine relating to the increasing number of cases of cancer and diabetes and the lack of effective pharmacological agents for such diseases are encouraging the scientific community to search for, acquire and study new bioactive substances of natural origin with potential medical and pharmacological applications. This distinctly rising trend is reflected in numerous publications, e.g., Newman et al. [20], Ngo et al. [21], and Yuan et al. [22]. Natural products are the source of some 50 % of all pharmaceuticals currently applied in medicine [23].

Products of plant [24,25], insect [26], and fungal [23] origin are considered good sources of such compounds, as are those produced in synergistically interacting structures observed, e.g., in ant nests [27]. Among the natural sources of pharmacological agents, insects and their products play an important role in ethnomedicine, including traditional Chinese medicine [28]. Based on their very interesting antibacterial, immunostimulative, diuretic, antirheumatic, and anticancer properties, these preparations are becoming an important aspect of classical modern medicine [9,29]. The main aim of the present paper was to characterize new water extracts of a substrate, popularly referred to as carton, obtained from *Lasius fuliginosus* nests constructed in both the above- and below-ground parts of deciduous tree trunks. For the first time, products containing metabolites produced by synergistically interacting elements, i.e., ants, plant structure material, and the microbiome of the structures built by these insects, were isolated and characterized in terms of their quality and bioactive properties. To date, only a very few scientific reports have been published on this subject. One of the few examples relates to anticarcinogenic terpenoid substances isolated from the ant nests in myrmecophyte plant *Myrmecodia pendens* [9]. Extracts from biological material can be prepared by various methods. Due to the richness of plant metabolites and their numerous applications, the methods of extraction from plant material, such as, for example, Pressurized Liquid Extraction (PLE) or the use of enzymatic hydrolysis, are quite extensively described [30,31]. The biological material used in this study was heterogeneous and sonication in water proved to be the most effective extraction method (Section 4.3)

The coevolution of ants and the fungi they cultivate stretches back for some 50 million years [32], during which time extremely complex, unique mutualistic relationships were established between them [33]. The fungi and ants became highly specialized, this emerging from the necessity to diversify, as it were, the cultivation by ants of both fungi and the microorganisms producing antipathogenic substances [34].

This study revealed clear differences in the structure (granulation of individual fractions, color) of the nests collected from different habitats (Figure 1C–H); they relate not only to the species of a particular tree, but also to its state of health. Carton samples E2 and E6 are brownish-red in color. This is probably due to the infection of the host trees by arboreal fungi leading to the formation of a brownish-red rot. In both cases, the willows in which the ant nests were found (E2-*Salix alba*, E6-*Salix fragilis*) had been attacked by the fungus *Laetiporus sulphuraeus*, which causes rapidly proliferating brown cubical rot of the tree heartwood [35,36]. This may be the reason why the carton produced by ants from such wood has this color. 

By cultivating fungus plantations in their nests, ants create an optimal and stable environment (darkness, appropriate temperature and humidity, etc.) in which various pathogenic organisms can flourish [37,38]. Despite this, their cultivations are very clean and resistant to infection, pre-conditions for the healthy growth and development of the whole ant colony: these insects have developed a range of methods protecting their nests from infection. Apart from selective cleansing by workers, ants utilize antimicrobial substances that they secrete, and coexist with symbiotic bacteria, including *Pseudonocardia*, *Streptomyces*, and *Amycolatopsis*, which themselves produce antibiotics [8,39,40].

The carton nest of jet black ants *Lasius fuliginosus* consists of three main components: fragments of wood with mineral particles, honeydew, and the fungus that glues everything together, which according to current knowledge is *Cladosporium myrmecophilum* [41,42]. The question of the ants’ glandular secretions has not yet been resolved: the chromatographic analyses by Maschwitz and Hölldobler [41] showed that they were not a constituent of the carton. On the other hand, the experiment carried out by Brinker et al. [12] suggests something quite the opposite: they showed that in the presence of an extract produced from body parts of *L. fuliginosus*, the fungus permeating the carton nest was growing faster. They also performed a similar experiment on a pathogenic fungus occurring in the nest, against which strong inhibitory activity was recorded. These experiments clearly demonstrate that the ants utilize their glandular secretions for managing the fungal plantations, which is why these substances are present in the nest. 

There are still gaps in our knowledge regarding the bacterial nest symbionts of *L. fuliginosus*: only a few papers have been published on the bacteria associated with this ant species [10,12]. The total microbial (TMA) activity determined in the present work for all the extracts showed the highest level of released fluorescein in E6 and the lowest in E2. The values recorded for E3–E5 were at a similar level. This suggests a close relationship between microbiome development and environmental conditions in the carton produced by the ants. The biologically extremely complex system has coevolved over millions of years as a result of the interaction of ants, fungi, and bacteria, whereby the mutual relationships among the microorganisms in the nest occur at both the antibiotic [43] and biotic levels [44]. These interrelationships are also reflected in the substances produced in these nests by each of the organisms and additionally derived from plant building material. It was for this reason that the carton employed in the present research was taken directly from the environment—no single substances were isolated from this material. This was treated holistically, together with all of its constituents, as a single, natural product, the aqueous extract of which was used in the subsequent study. 

Comparison of biochemical properties revealed clear differences between the tested extracts from the different habitats. This is well exemplified by the biochemical parameters of the extracts, such as proteolytic activity, and the contents of proteins, sugars, and phenolic compounds. Comparison of the extracts showed that proteolytic enzyme activities were the highest in E3 and E4, and the lowest in E5. As is well-known, proteases are directly involved in the cellular protein turnover of living organisms [45]. The highest protein concentrations were recorded in extracts E1 and E6; interestingly, the relevant values in the preparation obtained from the underground nest (E4) were distinctly lower than the others. The results showed that extract E4 clearly differed in both composition and biological activity from the other ones. It is worth noting at this juncture that this was the only underground nest among the six studied, situated as it was under the roots of a silver birch *Betula pendula*, with the entrance in the soil at the base of the trunk. The other five samples were taken from sites at least 1 m above the ground, in tree hollows or cracks in tree trunks. Basically, an underground nest serves ants as an overwintering site [41]. Our results appear to confirm that the location of the nest has a key impact on the properties of the extract, because the associated different environmental conditions can significantly affect, for example, the production of secondary metabolites by *Streptomyces* [46]. The concentration of phenolic compounds and antioxidant potential in extract E4 were found to be the lowest. 

Since the extracts were characterized by antioxidant properties and the presence of phenolic compounds, proteins, and polysaccharides, it was also decided to evaluate their cytotoxic potential. One of the most malignant human cancers is melanoma, which is why it was selected for further analysis. Melanoma (melanoma malignum) is a type of malignant tumor derived from melanocytic neuroectodermal cells; it is characterized by unusual dynamics and is highly invasive. The situation is further aggravated by the fact that, unfortunately, most people still have little awareness of either the disease factors or of melanoma prevention [47,48,49,50]. In addition, malignant melanoma has a high metastase potential, which is extremely difficult to treat pharmacologically. This means that a melanoma transforms relatively quickly from being a disease of a local nature (activating within a specific field on the skin) into a generalized/diffuse form. In addition to spreading over the surface of the skin, a melanoma can also grow deep into it. If it manages to penetrate the skin barrier, it can enter blood vessels, and from there, the whole body [48,51]. The treatment of such a highly metastatic tumor is difficult, hence the need for new preparations that are effective against the cancer, but harmless to healthy cells. For this purpose, substances of natural origin with anticancer potential are being sought. An example of potentially active substances against melanoma cells, derived from natural sources, are the high- and low-molecular weight bioactive subfractions isolated from the mossy maze mushroom *Cerrena unicolor* by Statkiewicz et al. [52], who demonstrated that all the fractions exhibited activity against mouse melanoma B16-F10 cells. Our analyses have shown that all the extracts (E1–E6) possess antiproliferative activity against melanoma cells as well as a high antioxidant potential. 

As already mentioned, the carton used in this work as the source of bioactive substances is a complex biological system in which plant, insect, fungal, and bacterial components participate. Extracts E1–E6 contained numerous metabolites which may have originated from both the plant material as well as the other components. The medicinal properties of willows have long been known. The bark and shoots are used as anti-inflammatory and analgesic medications [53,54]. Apart from their content of salicylic acid, willows contain a range of other compounds, like other salicylates, flavonoids and polyphenols [55], and preparations made from ground parts of plants have exhibited antioxidant and inhibitory activity against acetylcholinesterase [56]. The results of our study show that the most beneficial anti-proliferative effect was achieved with extract E2, obtained from a willow. This preparation displayed the highest sugar content and antioxidant activity, which may be directly related to its action against cancer cells. The biological properties of polysaccharides or phenolic compounds, which are well-known free radical scavengers, have been frequently described in the literature [57,58,59,60,61]. 

Fungi are a well-known source of bioactive substances of major biomedical importance. The substances obtained from them, e.g., phenolic compounds, exhibit anticancer, antibacterial, immunostimulatory, and antioxidant properties [59,60,62,63,64,65]. It seems, therefore, that apart from plants, the substances produced by *Cladosporium myrmecophilum* fungi present in the carton make a significant contribution to the unique properties of the extracts, especially E2. The secondary metabolites of fungi can modulate processes such as apoptosis, angiogenesis, and metastasis, and can also regulate cell cycles, transduction cascade signals, and immune responses [66,67,68]. Kuriyama et al. [69] showed that low molecular weight polyphenols from *Inonotus obliquus* inhibited not only DNA polymerase and DNA topoisomerase, but also the proliferation of HCT116 colon cancer cells. Other authors reported cytotoxic and mutagenic effects of *F. trogii* and *C. versicolor* extracts on the HeLa cervical cancer cell line and human fibroblast cells [70]. Bioactive proteins are another important type of functional cytotoxic fungal components. Liu et al. [71] isolated a non-immunological xylose-specific lectin protein (28.8 kDa) from *Xylaria hypoxylon* fruitbodies with potent antimitogenic and antitumor activity.

Insects have an extremely efficient immune system based on the production of potent antimicrobial peptides (AMP) in response to the appearance of a pathogen [72,73]. It turns out that the peptides produced by insects and other arthropods also exhibit evident activity against cancer cells, including those of malignant melanoma, as demonstrated by Rodrigues et al. [37], among others. These authors tested the spider peptide gomesin on melanoma cells B16F10-Nex2 via local administration on affected mice. The production of immune peptides in holometabolous insects, including ants, takes place in the fat body and the epithelia [74]. Consequently, they proliferate throughout the body and can penetrate the carton along with other ant secretions. This could have a significant impact on the antiproliferative and cytotoxic properties of the tested extracts against melanoma cancer cells, with a simultaneous lack of negative impact, or even nutritional action on normal cells. Hasanuddin et al. [9] investigated the use of an extract derived from ant nests built inside the shoots of a plant from the species *Myrmecodia pendens.* These authors were able to demonstrate its apoptotic action against ovarian cancer cells SKOV-3 in concentrations of 200–600 μg/mL. In our study, concentrations as low as ca. 100 μg/mL of an extract from nests of *L. fuliginosus* exhibited quite a significant level of antiproliferative activity. 

The results of our FTIR analysis are in agreement with those of the biochemical analysis. The extracts exhibited a high probability of polysaccharides and lignin-derived phenolic compounds (acids or aldehydes) being present in all the tested samples of aqueous extracts from anthills. These data are in good agreement with the results published by Kristiansen and Amelung [75]. They found that abandoned anthills contain large amounts of lignin-derived phenols (acids rather than aldehydes) and cellulosic and non-cellulosic polysaccharides, which probably reflected the collection of woody debris for the nest construction. Interestingly, our extract E2 (and to some extent E1, E3, E4, and E5 as well) exhibited a band at 1512 cm^−1^. This band was reported to appear as a result of the formation of Fe(III)-syringic acid complexes [18]. Iron oxide (Fe_2_O_3_) was reported to be present in anthill clay in concentrations as high as 9.41% [76]. Iron oxide, being highly soluble in acids, could dissolve in formic acid (the product of the poison glands of ants), and Fe^3+^ ions could form complexes with lignin-derived phenolic compounds. It was shown that some polyphenols may act as scavengers of reactive oxygen species and chelators of prooxidant metals (such as iron and copper) [77]. It is therefore possible that the presence of metal–phenolic acid complexes in our samples of anthill extracts reflects their high antioxidative potential.

Having analyzed the biochemical parameters and the action against melanoma cells, we tested the antiproliferative activity of the most effective extract E2 using ECIS. The search for new drugs and the screening of bioactive compounds are often performed in cell-based test systems in order to reduce costs and save time. An innovative method for monitoring live cells is the real-time analysis of selected electrical parameters, i.e., cell membrane capacitance, resistance, or impedance. By culturing cells on the electrode surface, the method of monitoring these parameters may directly provide detailed information about the cellular activity in that parameters are measured and methods of multiple marking are eliminated, thus facilitating non-invasive examination of cellular properties in real time. Additionally, electric cell-substrate impedance sensing (ECIS) measurements provide information about temporal changes in cell–cell-contacts not available for single cell observations. Dead cells are easily detached from the electrode surface. The changes in cell adherence to the electrode surface result in changes in ECIS measurement data; cell adherence is therefore reflected by ECIS data [78]. When impedance measurements are performed on intact cells, these act as a resistor and capacitor connected in parallel owing to the characteristics of their membranes. Here, resistance represents the opposition to current flow, whereas capacitance (C) describes the separation of electric carriers at the insulating bi-layer of the cell membrane that causes cell polarization. Capacitance provides an overall measure of electrode coverage. 

As a result, the different behavior of the cells after seeding, adherence, proliferation, and their reaction to substances added to the substrate, produce a change in the impedance. From the measurement of capacitance, it can be concluded that higher frequencies are best suited for cell spreading because they increase cell coverage of the electrode. At high frequency ranges, measurement is more sensitive to changes in cell attachment and spreading at the electrode [79]. Nevertheless, according to Wegener et al. [80], 10–40 kHz is the main frequency range used for measuring impedance. The results of our work concur with the studies of Hofman et al. [81] in that impedance readings at 32 kH are the most robust indicator for cytotoxicity. The electrical impedance is defined as the opposition to the electrical current within a circuit. In systems utilizing direct current, the impedance is simply the resistance, but in systems utilizing alternating currents, the changing electric and magnetic fields create additional and varying opposition to the applied current [82]. This phenomenon has also been observed for Vero and melanoma cells. Manifestly lower impedance values in the case of melanoma compared to normal Vero cells, after treatment with extract E2 at a concentration of 250 μg/mL, indicate the possible antitumor effect of the test substance, whilst negatively affecting the Vero cells. These results correlate with data obtained in the MTT test, where the antiproliferative effect of the test substance was demonstrated.

In summary, our research results have shown that ant nest water extracts may well offer a new alternative and effective source of pharmaceuticals with chemopreventive and antioxidative potential. It should be emphasized that very little research has been conducted to date regarding the antitumor activity of such preparation. It therefore seems highly probable that synergism is responsible for the combined activity of all the substances contained in carton, as in the case of the activity of the secondary metabolites produced by the symbiont microbial communities of leaf-cutting ants *Acromyrmex* [27]. The effects of this unique synergistic action, presented in this work on the basis of extracts from ant nests, are proposed for the first time as a new, alternative source of bioactive, i.e., anticancer, antioxidant, and antibiotic substances, and may turn out to become important in the control of various human and animal diseases.

## 4. Materials and Methods

### 4.1. Environmental Conditions of the Biological Material 

The biological sources of the extracts (E1–6) used in this work were the nests of jet black ants *L. fuliginosus* constructed from a characteristic material commonly referred to as carton. The study material consisted of nest fragments taken from different tree species at six sites, each of a different habitat type (Table 3).

### 4.2. Visualization of the Ant Nest Carton Structure 

The microstructure of the ant nests was examined and recorded using an Olympus DP21 digital camera mounted on an Olympus BX63 compound microscope or VEGA3 TESCAN SEM (Figure 1B–J). Fresh samples in the field were recorded using Panasonic DMC-TZ60 digital camera (Figure 1A1). For the SEM techniques, samples were dried at the critical point of CO2 using an Emitech K850 Critical Point Dryer. The dried samples of carton were coated with a layer of gold using an Emitech K550X Sputter Coater. Thereafter, the samples were placed directly in the SEM chamber for examination.

### 4.3. Extract Preparation 

Around 500 mL of material obtained from the six types of ant nests was taken from each nest; this was an optimum quantity for research use, and not so much that the ants could not quickly make good the loss. The person sampling the material wore rubber gloves, and the material was placed in sterile, hermetically sealable containers. Unwanted fragments of wood and ants (adults and larvae) were removed in the field using sterile tweezers. This preliminarily sorted material was sealed in containers and taken to the laboratory, where it was first put in a freezer. For the analyses, suitable portions of the frozen material were placed in sterile test-tubes using sterile tweezers. Portions of ant nest carton were suspended in MQ redistilled water at a 1:10 weight-to-volume ratio and mechanically pre-treated using a blade homogenizer. The mechanically pre-ground biological material was sonified (80% amplitude, 4-min cycle-30 s pulse/30 s cooling the preparation in ice), filtered through a Miracloth membrane, and centrifuged (10,000 g for 15 min at 4 °C). The supernatant was freeze-dried and used in the subsequent research as a preparation (extract E1–E6). For all biochemical analyzes presented in the paper, an aqueous solution of the obtained samples in a concentration of 1 mg/mL was used.

### 4.4. Biochemical Analysis of Extracts

#### 4.4.1. Estimation of Total Carbohydrates (TC), Proteins, Phenolic Compounds (PC), Relative Level of Superoxide Anion Radicals (SOR) and Proteolytic Activity 

The concentration of total carbohydrate in the extracts was estimated using the phenol-sulfuric acid assay (D-glucose was the standard) [83]. The protein content of the extracts was determined using the method based on Coomassie brilliant blue (G-250) dye with bovine serum albumin as standard [84]. Phenolic compounds (-hydroxyl, -methoxy phenolic acids) were determined quantitatively with the DASA test (diazosulphanilamide (SA) was the reaction substrate) [85]. Absorbances were recorded at 500 nm and compared with the data from the calibration curve (y = 6.85x − 0.0218, R2 = 0.999). Nitrotetrazolium blue (NBT) was used to assess the relative level of superoxide anion radicals in the extracts. Characteristic of this method is the use of an alkaline reaction medium, which prevents precipitation of dark-blue formazan during the 40 min incubation time [86]. A fluorescent micro-method based on the digestion of BSA-BIODYPY heavily labelled bovine serum albumin (Invitrogen DQ™ Red BSA) substrate in different pH ranges (3.0, 7.0, 8.0) was used to determine the proteolytic activity. 50 μL (50 μg/mL) of substrate and 50 μL (1 mg/mL) of analyte were added to 100 μL of McIlvaine buffer of the appropriate pH. The increase in fluorescence was measured over 30 min at 590 nm excitation and 620 nm emission using a Tecan Infinite m200 pro plate reader. The specific activity was expressed as the increase in relative fluorescence units per min per μg of protein. 

#### 4.4.2. Antioxidative Capacity

The antioxidative properties of the six extracts (E1–6) were determined according to the procedure adopted by Paduch et al. [87], based on the DPPH (1.1-diphenyl-2-picrylhydrazyl) reaction substrate. Ascorbic acid, a strong antioxidant, at concentrations from 6.25 to 800 μg/mL, was the positive standard in this method. The ABTS radical-scavenging activity assay was performed according to van den Berg et al. [88], Duo-Chuan [89], and Re et al. [90] with modifications. The 7.4 mM ABTS stock solution in MQ water, activated by 2.6 mM potassium persulphate, was incubated in the dark for 16 h at room temperature. The stock solution was subsequently diluted with phosphate buffered saline (PBS) at pH 7.4 to an absorbance of 0.7 AU at 734 nm. The assay was performed in a 48-well microtitre plate containing 10 μL μL of the test sample and 990 μL of the ABTS radical solution. The radical scavenging activity was calculated from the ascorbic acid calibration curve. 

#### 4.4.3. Determination of the Total Microbial Activity Level (TMB)

Spectrophotometric detection of TMB was conducted on the basis of fluorescein diacetate (FDA) hydrolysis, which is directly related to the respiratory activity observed in the tested biological sample. After 3 h incubation, the reaction was terminated by the addition of acetone to the reaction mixture [91]. TMB was expressed as mg of released fluorescein/kg/1 h.

#### 4.4.4. FTIR Analysis 

The FTIR-ATR spectra were taken in a Vertex 70 spectrometer coupled to a Hyperion 3000 microscope equipped with MCT detector and germanium crystal (Bruker, USA) (64 scans, resolution 4 cm^−1^). Samples were dried before the analysis in an exicator to remove traces of unbound water. Spectra were analyzed by OPUS 7.0 software (Bruker, USA).

### 4.5. Analysis of Anticancer Activity of Samples

#### 4.5.1. Cell Lines and Culture Conditions

The Vero cells were obtained from ATCC (ATCC CCL-81™) and cultured according to the manufacturer’s instructions. The cells were maintained in ATCC-formulated Eagle’s Minimum Essential Medium supplemented with 10% FBS Good HI (fetal bovine serum) and antibiotic-100 IU/mL Streptomycin/Penicillin/Amphotericin B. The A-375 [A375] (ATCC^®^ CRL-1619™) tumor cells were maintained in ATCC-formulated Dulbecco’s Modified Eagle’s Medium. Other additives included 10% FBS Good HI (fetal bovine serum) and an antibiotic-100 IU/mL Streptomycin/Penicillin/Amphotericin B. The process was carried out in a Galaxy 170R incubator under controlled growth conditions, constant humidity, and 5% CO2 air saturation.

#### 4.5.2. MTT Assay

The method is based on the ability of mitochondrial succinate dehydrogenase to reduce yellow tetrazolium salt (MTT) to purple formazan crystals. As this activity is performed by live cells, absorbance is directly proportional to the quantity of live cells [92,93]. After multiplication and stabilization of the cells (approx. 7–14 days), when the culture reached at least 75% confluence, the next stage of testing was culturing the cells (cell suspension with a density of ~1.2 × 10^5^ cells/mL) with the preparations each at different concentrations (2.5, 25, 50, 100 and 250 μg/mL) for 24, 48, and 72 h. After 24, 48, or 72 h, cells from each variant were harvested by trypsinization and transferred to the MTT Cell Proliferation/Viability Cell Assay: BIOKOM analysis–96-well plates in triplicate–of 100 μL cell dilutions of 1 × 105 cells/mL per well (optimal density determined experimentally). The control contained only cells (Vero or A-375) without the test preparation. The plates were incubated for ca. 24 h (left overnight), after which 10 μL MTT reagent was added to each well. The plate was returned to the incubator for 2 to 4 h until a purple color appeared. When the purple precipitate was clearly visible under the microscope, 100 μL of detergent reagent was added to all wells. The plate was covered and incubated at room temperature overnight in the dark. The plates were read using a Microplate Reader, and absorbance at 570 nm was measured (MULTISCAN FC Thermo Scientific).

#### 4.5.3. Electric Cell-Substrate Impedance Sensing (ECIS)

The ECIS system Zθ instrument (Applied Biophysics Ltd., Troy, New Jersey, USA), supplied by ibidi GmbH, was used to measure the impedance. It contained two separate units: the station controller Zθ, located outside the incubator, and a docking station containing two 8-well plates, which was placed in the incubator space. The electrodes used were 8W10E, which comprised 8 wells and 10 active electrodes in each well. The ECIS electrodes were placed in a holder plate in a humid incubator (Galaxy 170R) at 37 °C and 5% CO2. Prior to inoculation, the arrays were incubated for 24 h with Eagle’s Minimum Essential Medium (Vero) and Dulbecco’s Modified Eagle’s Medium (A375) in the Galaxy 170R incubator overnight. Following stabilization, the array was removed from the array station and inoculated with cells. Inoculation of arrays was carried out using 600 microlitres per well of cell suspension ~1.2 × 10^5^ cells/mL. The extract was added to inoculated wells at the final concentration of 250 μg/mL. After cell manipulation, the matrix holder was placed in an incubator and real-time measurements were initiated. The maximum response for Z, R, and C occurred at different frequencies. The default optimal frequencies were used in this study: Resistance (R) 4000 Hz, Impedance (Z) 32,000 Hz, Capacitance (C) 64,000 Hz. The changes in cellular behavior in response to the compound were recorded as impedance signals and the data obtained processed through ECIS software. When cells are stimulated to change their function, the accompanying changes in cell morphology alter the impedance. The data generated is impedance versus time.

### 4.6. Statistical Analysis 

The results of the experiments were expressed as mean ± SD from three experiments (*n* = 3). The results were analyzed by ANOVA, and means were compared using Tukey’s post hoc test. All calculations were conducted using the STATISTICA (StatSoft Polska). Only values with a significance of *p* ≤ 0.05 are reported as different.

## Figures and Tables

**Table 1:** Concentration of proteins, phenolic compounds (PC), total carbohydrate (TC), relative level of superoxide anion radicals (SOR), and total microbial activity in the extracts obtained from the ant nest carton. Values with different letters (a–e) are statistically significant (*p*  <  0.05).

	E1	E2	E3	E4	E5	E6
Protein (μg/mL)	92.20 (12.4) a	78.90 (13.18) a	79.89 (12.34) a	27.13 (2.13) b	83.30 (3.85) a	97.65 (6.72) a
Total phenolic compounds (mM)	0.13 (0.00) a	0.20 (0.04) b	0.14 (0.03) c	0.05 (0.00) d	0.14 (0.02) c	0.11 (0.01) e
Total carbohydrates (μg/mL)	145.07 (38.5) a	241.87 (60.63) a	162.46 (27.14) a	144.60 (30.51) a	144.01 (27.6) a	172.2 (26.44) a
Relative level of free radicals (absorbance 560 nm)	0.54 (0.04) a	0.44 (0.06) a	0.24 (0.05) b	0.10 (0.02) c	0.54 (0.03) a	0.27 (0.4) b
Total microbial activity (mg/kg/h)	0.42 (0.00) a	0.38 (0.00) b	0.57 (0.01) c	0.56 (0.01) c	0.47 (0.01) d	0.62 (0.03) e
ABTS radical-scavenging (μg/mL ascorbic acid equivalent)	188.96 (3.01) a	313.67 (3.35) b	164.69 (17.63) a	96.12 (7.27) c	70.97 (3.58) d	132.15 (9.31) e
DPPH radical-scavenging (μg/mL ascorbic acid equivalent)	176.42 (1.42) a	202.66 (6.01) b	128.80 (2.36) c	98.75 (9.2) d	146.24 (1.36) e	150.05 6.92) e

**Table 2:** IC_50_ values of extracts E1-E6. The IC_50_ on the Vero line could not be determined for E1, E2, E4, E5 and E6, as their cell viability was >50%.

Sample	E1	E2	E3	E4	E5	E6
IC_50_ [µg/mL]						
VERO	-	-	2034	-	-	-
A-375	77.17	123.5	90.75	0.01	63.92	607.5

**Table 3:** Description of the habitat conditions of the ant nests used as the source of biological material for extracts E1–E6.

Extract	Description of Habitat Conditions
E1	The samples were obtained from a pedunculate oak (*Quercus robur*) situated in dry mixed forest; the ant nest was in a hollow. This solitary tree was growing in a quite fertile, fairly moist, dense tree nursery–scrub with birch, aspen, and oak undergrowth. The oak tree in question was alive, but with rotting heartwood. The hollow was a longitudinal section of rotting wood at the base of the trunk, closed off from above by live tissue, tapering upwards from the base, where it was at its widest ca. 60 cm; the crevice was more than 1 m in overall height.
E2	The source of the biological material was a white willow (*Salix alba*) in a riparian poplar-willow forest in the valley of the River Wieprz; the ant nest was in a tree hollow. The willow was growing in very dense scrub passing into riparian forest; immediately adjacent were hazel (*Corylus avellana*) and elder (*Sambucus nigra*). The tree was alive, partially pollarded; the trunk had a large open hollow with rotting wood extending from the trunk base to a height of ca. 1.5 m above the ground; inside, the hollow was partially empty, but the upper rear part was filled with decaying wood and a large jet black ant nest.
E3	The nest was located in a hollow in a crack willow (*Salix fragilis*), in the valley of the River Wieprz. The willow was growing by a dirt road leading to the river; the adjacent scrub on the moist substrate of the valley was overgrown with hop (*Humulus lupulus*), with numerous white and crack willows nearby. The tree was bent, parts of it were dead, the trunk was covered with fruiting bodies of *Phellinus* polypores, and mistletoe (*Viscum album*) was growing on the branches. The hollow containing the nest extended more or less from the base of the trunk to a height of ca. 1 m, tapering upwards.
E4	The samples were obtained from a silver birch (*Betula pendula*) in mixed woodland; the nest was a subterranean one. The tree was growing in a low-density stand of mixed woodland with pedunculate oak, Scots pine (*Pinus sylvestris*) and birch, with hazel and alder buckthorn (*Frangula alnus*) in the underbrush, and a young pedunculate oak in the immediate vicinity. The subterranean nest was situated in a crevice between the tree’s roots some 30 cm from the trunk base; the opening of the crevice was ca. 20 cm in diameter, and overlain with twigs and leaf litter; this was removed while the sampling material was being collected, then replaced.
E5	The ant nest that was the source of the biological material was located in a hollow of a silver birch (*Betula pendula*) in an oak-hornbeam forest near Lake Długie. The tree, now dead, had stood at the edge of an oak-hornbeam forest, near a woodland bog. The hollow leading to the ants’ nest was at a height of ca. 80 cm above the ground. The birch had been blown down during an autumn storm, so a large amount of material could be collected. On the day the material was sampled, there were no ants in the nest.
E6	The samples were obtained from a crack willow (*Salix fragilis*) in a riparian poplar-willow forest in the valley of the River Wieprz; the nest was situated in a tree hollow. This solitary tree was growing in alluvial scrub in the close vicinity of a riparian forest community. The hollow was at a height of ca 1.5 m above the ground, with an opening ca 10 × 15 cm; the extensive nest was visible from the exterior *.

* The ant species studied here, *Lasius fuliginosus*, is not on the list of protected species in Poland, so research on it does not require a permit. The material was obtained from both unprotected areas (Rogóźno and Łańcuchów) and from the Polesie National Park (Pieszowola and the area near Lake Długie). The field studies in the Polesie National Park were approved by the Polish Ministry of Environment (field study permit number: DLP-III.286.148.2016.MGr; DLP-III.286.141.2016.MGr).
